# Cushing’s Syndrome Masquerading as Fibromyalgia: A Case Series

**DOI:** 10.7759/cureus.68181

**Published:** 2024-08-30

**Authors:** Hind H Al Samsam, Fahad Fazal, Shamma Al Nokhatha, Asma AlJaberi, Abdelrahman Adam, Said Huwaijah, Wael Bekhit

**Affiliations:** 1 Internal Medicine, Mediclinic Al Ain Hospital, Al Ain, ARE; 2 Rheumatology, Mediclinic Al Ain Hospital, Al Ain, ARE; 3 Rheumatology, Tawam Hospital, Al Ain, ARE; 4 Endocrinology, Tawam Hospital, Al Ain, ARE; 5 Radiology, Mediclinic Al Ain Hospital, Al Ain, ARE; 6 Orthopaedics, Mediclinic Al Ain Hospital, Al Ain, ARE

**Keywords:** cortisol, iatrogenic cushing’s syndrome, adrenal gland adenoma, fibromyalgia, cushing's syndrome

## Abstract

Three young female patients with a history of generalized body pain were diagnosed with fibromyalgia. They visited several specialities which related patients' symptoms to their previous diagnosis of fibromyalgia and were treated symptomatically. These patients developed a multitude of clinical features including fractures, hypertension, abnormal weight gain, proximal myopathic pain and bruising. They were seen by rheumatologists whose assessment was that their clinical features were not entirely due to fibromyalgia and suspected that patients have a possible underlying endocrine cause. Patients were referred to an endocrinologist for further tests with suspicion of Cushing’s syndrome. Laboratory tests and imaging confirmed a diagnosis of Cushing’s syndrome. Two of them had adrenal adenoma and one had iatrogenic corticosteroid use. These cases emphasize the need for thorough clinical evaluation for patients who are thought to have fibromyalgia. Fibromyalgia is a diagnosis of exclusion.

## Introduction

Fibromyalgia is a chronic functional neurosensory disorder characterized by diffuse musculoskeletal pain, fatigue, and insomnia [1]. The exact cause is yet to be understood and the diagnosis relies solely on the patient's history as physical examination, imaging, and laboratory tests are usually normal making it a diagnosis of exclusion.

Cushing’s syndrome is an endocrine disorder caused by an increase in cortisol level in the body due to either exogenous glucocorticoid administration or endogenous overproduction of cortisol due to adrenal adenoma, pituitary adenoma, or ectopic paraneoplastic foci [2].

Patients may present with central obesity, easily bruised skin, purple abdominal striae, osteoporosis and pathological fractures, secondary hypertension, hyperglycemia, fatigue, and proximal muscle weakness.

We herein report three cases of patients who had diffuse muscle pain and were misdiagnosed as fibromyalgia without ruling out endocrinological causes such as Cushing’s syndrome which they were found to have.

## Case presentation

Case report 1

**Figure 1:**
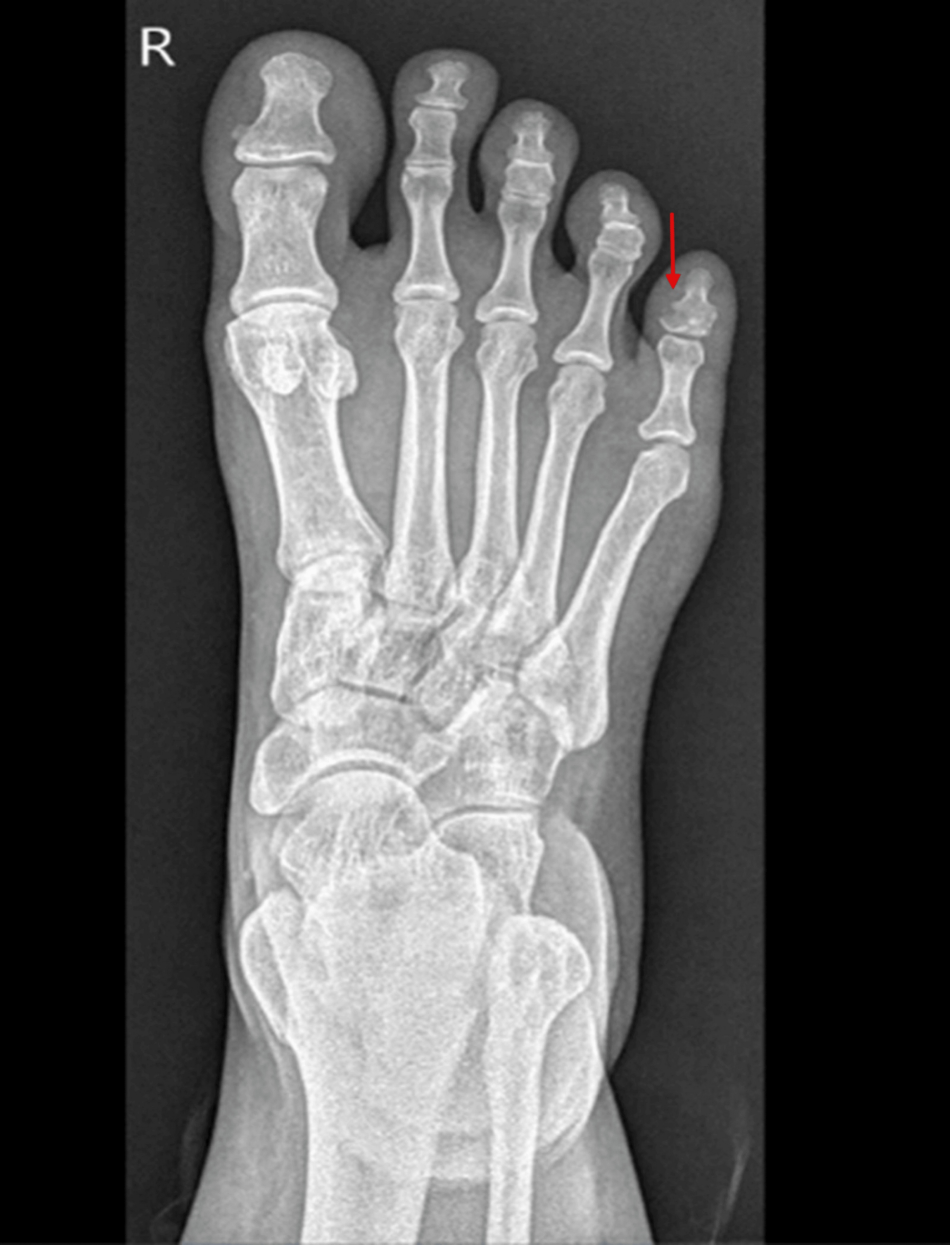
X-ray of right foot showed fracture at the distal phalanx of fifth toe with suspected intra-articular extension

A 38-year-old Egyptian female with a history of fibromyalgia presented to the urgent care in November 2020 with right little toe pain and swelling after hitting it against the wall. She had a fracture of the distal phalanx of the fifth toe (Figure 1) and was managed conservatively.

In January 2022, she presented to her gynaecologist with headache, body swelling and was found to be hypertensive (156/105mmHg). She was referred to cardiology for management of hypertension, who recommended keeping a blood pressure (BP) diary with one-week follow-up as her BP was high on one occasion only.

In May 2022, she visited an internist because of easy bruising for six years in both lower limbs and history of bleeding following dental procedure. She was also complaining of gaining weight (15 kg over seven months). Investigations including coagulation profile, serum electrolyte, blood glucose, liver enzymes, and autoimmune antibodies were ordered, and they were normal. Patient was reassured and was diagnosed as purpura simplex.

In September 2022, she had a visit to the cardiologist after she was diagnosed with hypertension in Egypt and was on ramipril (2.5mg) and torsemide (10mg). The cardiologist continued ramipril and discontinued torsemide. The cardiologist referred her to internal medicine because of her history of fibromyalgia, and review of her prescribed medications from Egypt which included duloxetine, hydroxychloroquine (HCQ), and melatonin.

**Figure 2:**
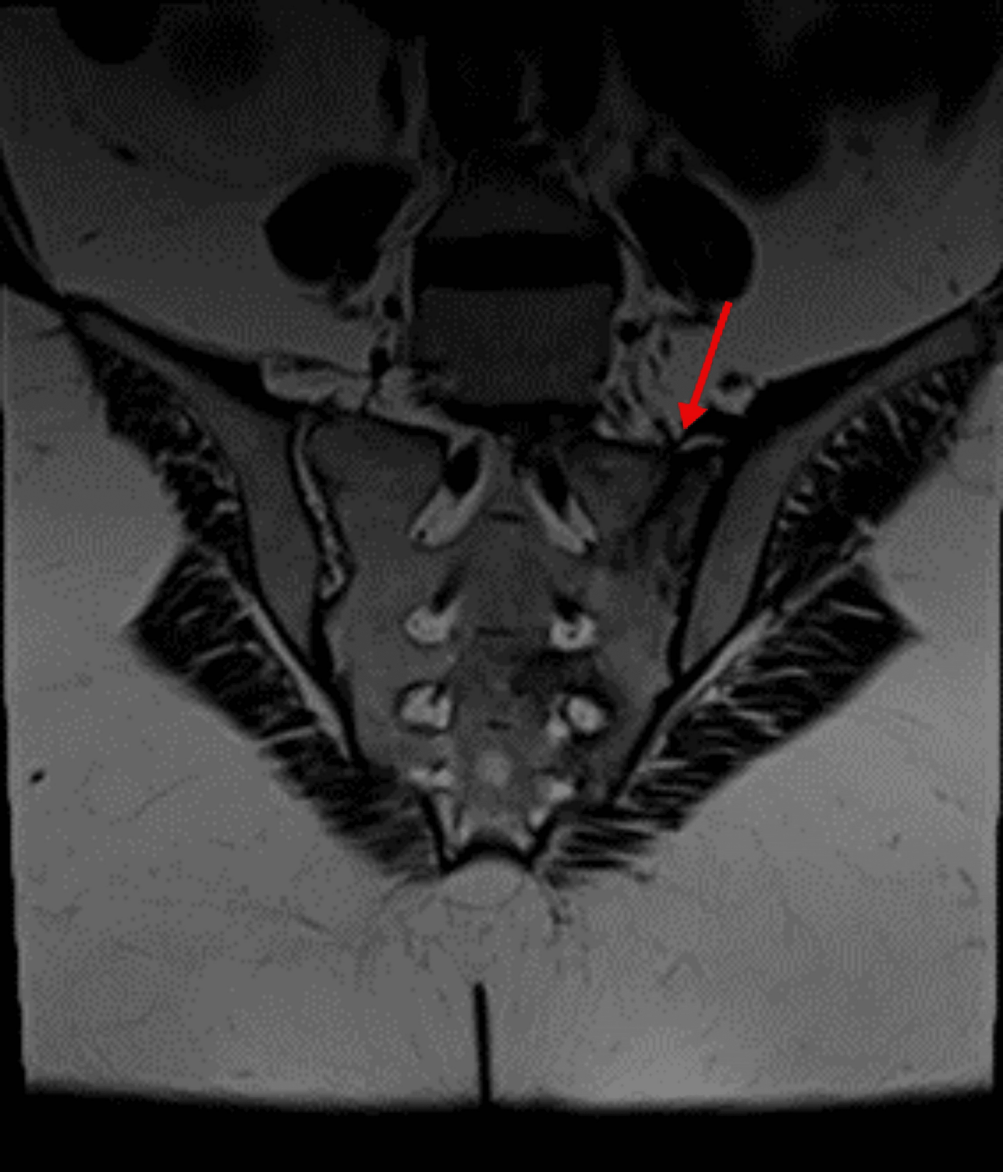
MRI sacroiliac joints showed left-sided linear sacrum fracture

She had multiple visits to internists between September 2022 and March 2023 with complaints of body swelling, generalized joint stiffness, hip pain, proximal myopathic pain when lifting arms or standing up with oral ulcers and small reddish-purple spots just beneath the skin’s surface most likely purpura simplex. Laboratory tests were ordered, and they showed she had low serum potassium and positive antinuclear antibody (ANA) titer (DFS-70 pattern). Also, she had negative rheumatoid factor (RF), extractable nuclear antigen (ENA) panel, antineutrophil cytoplasmic antibodies (ANCA) and anti-cyclic citrullinated peptide (CCP) with normal C-reactive protein (CRP) and erythrocyte sedimentation rate (ESR). She was given potassium supplements and magnesium. During her visits she was prescribed various medications for fibromyalgia including duloxetine, amitriptyline, and tramadol. She also developed back pain and her MRI of sacroiliac joints showed signs of left-sided linear sacrum fracture, crescentic subchondral edema in the right femoral head suggestive of avascular necrosis (AVN) and narrowing of L5/S1 intervertebral disc space with degenerative changes (Figure 2).

She then visited an orthopedic surgeon in April 2023 with back and right hip pain. The orthopedic doctor thought that her symptoms and signs were not entirely consistent with fibromyalgia, and she was referred to rheumatology for further review.

On rheumatology review she gave a history of whole-body pain, back pain, severe right hip pain, two fractures (left foot and sacrum), hypertension, hypokalaemia, amenorrhea for 18 months, weight gain (of 15 kg over seven months) and skin bruising. Laboratory tests showed negative autoimmune tests, low serum potassium, high alkaline phosphatase (ALP), normal parathyroid hormone (PTH), Mg, vitamin D and calcium. She was referred to internal medicine for low serum potassium, with suspicion of adrenocortical excess.

Her internist suspected Cushing’s syndrome as her physical examination showed that she was obese with florid purple striae on the trunk and arms in addition to proximal muscle weakness . He then ordered investigations that showed low adrenocorticotropic hormone (ACTH) using electrochemiluminescence immunoassay (ECLIA) of <1 pg/mL (normal range 7.2-63.3 pg/mL), and high serum cortisol using chemiluminescence microparticles immunoassay (CMIA) at 5 pm of 604.03 nmol/L (normal range 79.0-478 nmol/L). Her cortisol before 10 am that was collected at 9:02 am was 623.91 nmol/L (normal range 101-536 nmol/L). In view of these values, she was referred to the endocrinologist. Serum aldosterone, renin, and their ratio were all normal. 24-hour urinary cortisol was inconclusive because of low volume of urine. Luteinizing hormone (LH), follicle stimulating hormone (FSH), thyroid stimulating hormone (TSH), prolactin, metanephrines and normetanephrines were normal. It was planned to do overnight dexamethasone suppression tests (ODST), but patient travelled to Egypt.

**Figure 3:**
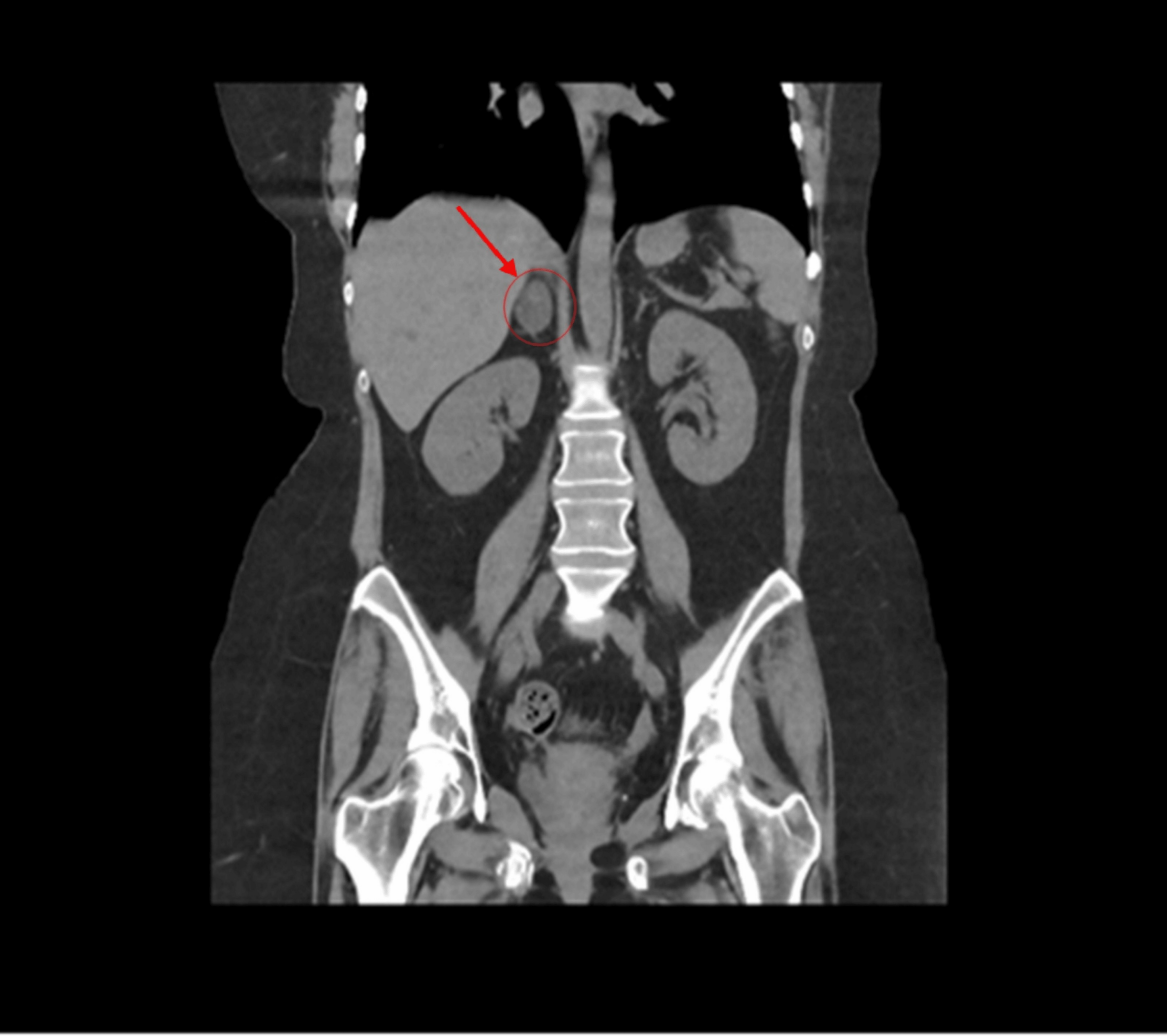
CT adrenal showed a 3.2×2×3 cm well-defined inhomogeneous density lesion arising from the junction between the arms of the right adrenal gland consistent with adrenal adenoma

**Figure 4:**
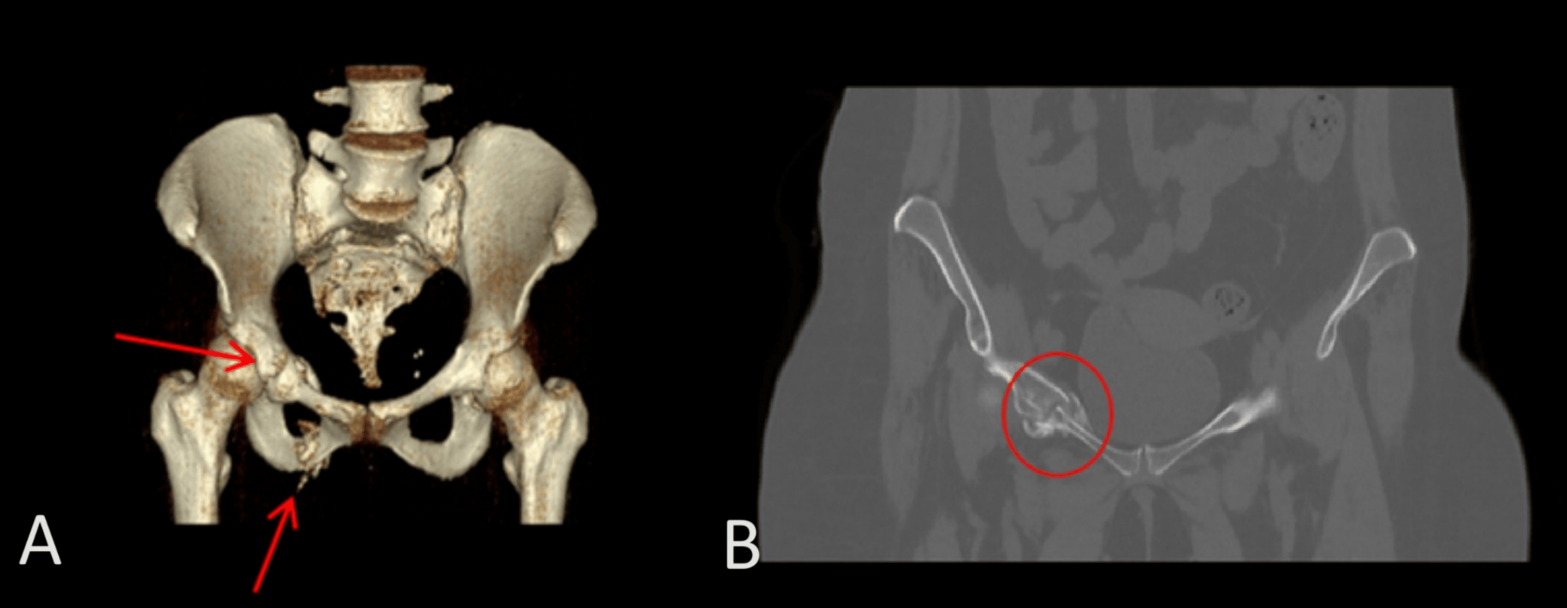
CT pelvis showed fragmented fracture at the inferior and superior ramus of right pubic bone associated with callus formation. Subcortical ill-defined lytic area is noted at the right humeral head surrounded with sclerotic reaction could be due to avascular necrosis (AVN)

**Figure 5:**
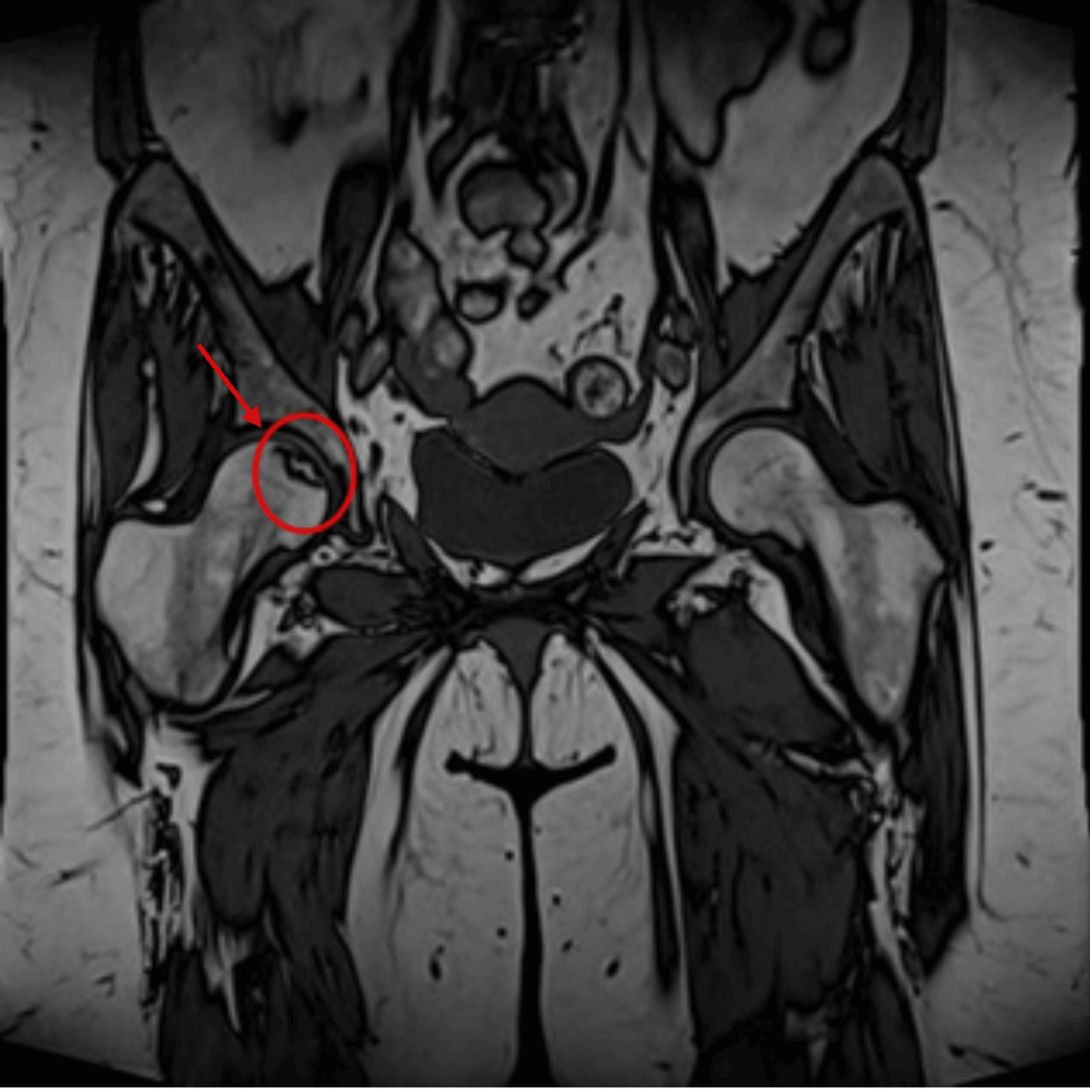
MRI of the pelvis showed subcortical geographic area at the right femoral head with inhomogeneous signal intensity (edematous and sclerotic changes) mostly due to avascular necrosis (stage II according to Ficat and Arlet classification)

CT abdomen showed a 3.2×2×3 cm well-defined lesion arising from the junction between the arms of the right adrenal gland showing inhomogeneous density with inhomogeneous enhancement after IV contrast administration with delayed washout, the maximum enhancement after the IV contrast administration at the portal phase about 55 Hounsfield units (HU) indicating a right adrenal adenoma (Figure 3). CT sacrum showed fragmented fracture inferior ramus of right pubic bone associated with callus formation and significant fragmented fracture lateral part of superior ramus of right pubic bone associated with callus formation (Figure 4). MRI hips showed avascular necrosis of the right femur head (stage II according to Ficat and Arlet classification) (Figure 5), which was treated with core decompression surgery.

**Figure 6:**
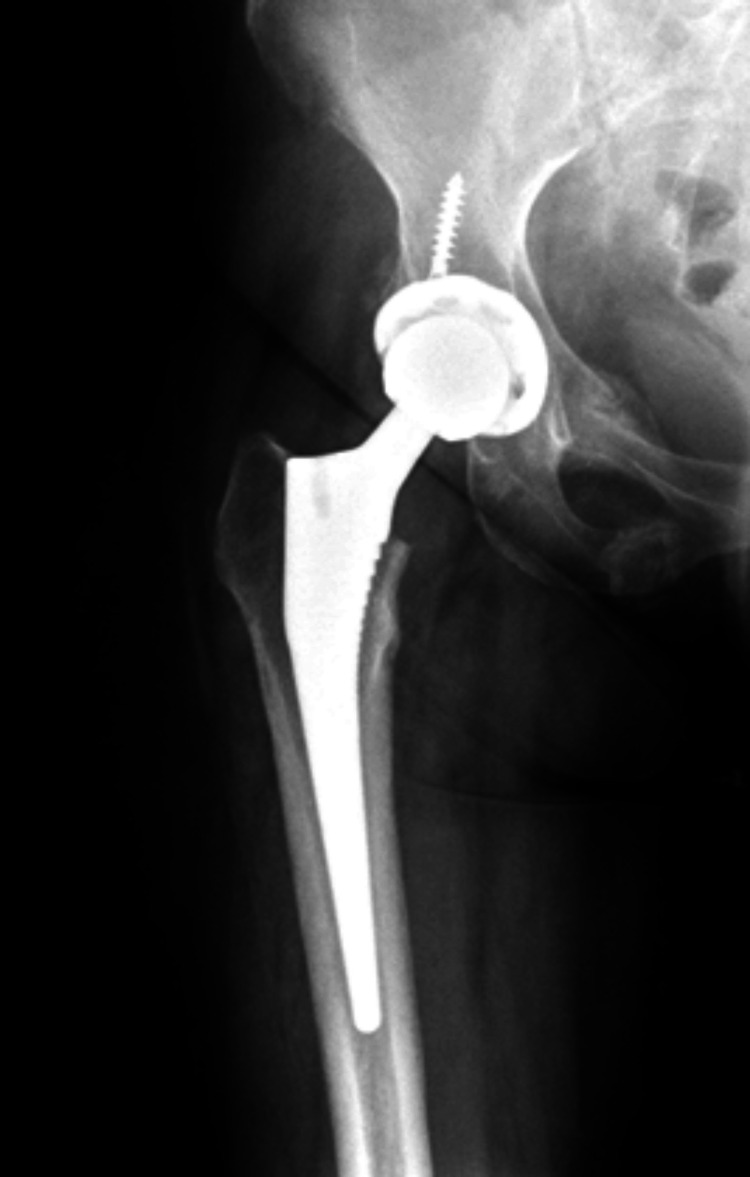
X-ray of the right hip joint showed signs of right hip joint replacement

She had the surgery to remove the adrenal adenoma in Egypt and histopathology confirmed the diagnosis. She was then started on corticosteroids as she had low serum cortisone levels after her surgery. Currently she is also taking duloxetine and calcium/vitamin D. She developed a fracture at the right femoral neck after a fall and had hip replacement in Egypt (Figure 6).

Case report 2

A 47-year-old Bangladesh female presented with a complex array of symptoms initially suggestive of fibromyalgia. The patient reported chronic widespread muscle and joint pain, with identification of approximately eight tender points during examination. These symptoms, coupled with fatigue, were initially thought to be fibromyalgia due to their nonspecific nature. Subsequently, the patient started to have multiple bone fractures. In total she had six fractures over one year including ﻿﻿fractures of the superior and inferior pubic ramus on the left side, right metatarsal bone fracture, fracture of the left proximal shaft of the fifth metatarsal, fractures of the shafts of the third and fourth left metatarsal. She has been reviewed by multiple physicians. A deeper look at her medical history revealed that despite the absence of overt Cushingoid features, she has several medical problems, including newly diagnosed hypertension and type 2 diabetes mellitus (hemoglobin A1C (HbA1C) 7.3%), raising the possibility of an underlying endocrine disorder. Psychiatric concerns involve a history of anxiety, insomnia, and major depressive disorder, with medication adjustments made independently. In addition, the patient reported irregular menstrual cycles, further complicating the clinical picture. Subtle signs such as unexplained central weight gain and telangiectasia prompted further endocrine evaluation.

Elevated morning cortisol levels and non-suppressed cortisol on an overnight 1 mg dexamethasone suppression test with high am cortisol, low am ACTH, ODST showed non-suppressed cortisol >400, and >500 on two occasions, and 24-hour urine free cortisol is high = 483 nmol (28-138). Adrenal CT without contrast revealed a well-defined heterogeneous isodense-to-hypodense lesion in the left adrenal gland, measuring 3.2 x 2.4 cm with a density of 16 HU, indicative of an adrenal adenoma. Imaging also identified old fractures of the left 10th rib and transverse processes of L1 and L4, which were previously undocumented and suggested underlying bone fragility.

**Figure 7:**
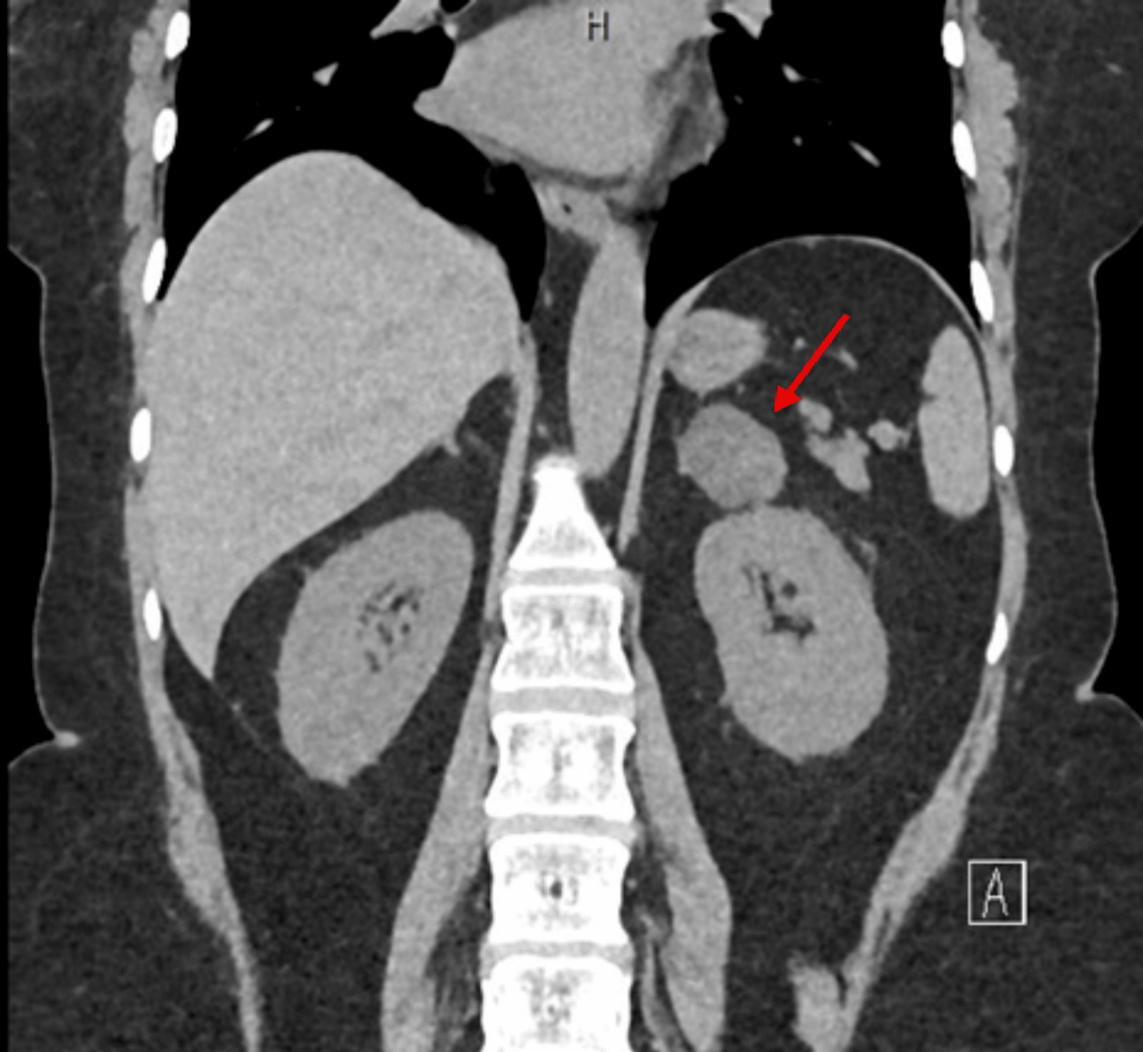
CT adrenal showed a 3.2 x 2.4 cm well-defined hypodense lesion in left adrenal gland

The combination of subtle endocrine symptoms, nonspecific musculoskeletal pain, and psychological components initially led to a misdiagnosis of fibromyalgia. However further endocrine investigation confirmed Cushing’s syndrome due to an adrenal adenoma (Figure 7).

The patient underwent successful laparoscopic removal of the left adrenal adenoma. Post-operatively, the patient developed adrenal insufficiency, necessitating a carefully managed hydrocortisone tapering regimen. Management of diabetes, hypertension, and psychiatric symptoms continued, with adjustments anticipated in response to changes in endocrine status post-adrenectomy. The patient was started on calcium and vitamin D supplementation to address the secondary osteoporosis.

Case report 3

A 35-year-old Emirati woman with a medical history of hypothyroidism, asthma, obstructive sleep apnea, scoliosis, secondary degenerative lumbosacral changes from a previous accident, and migraines sought consultation at the Department of Rheumatology.

She reported a two-year history of polyarthralgia, proximal muscle weakness, profound fatigue, and peripheral edema. BP was 148/88. Physical examination revealed a round face, dorsocervical fat pad, central obesity, and puffy hands and feet.

Laboratories revealed hemoglobin (Hb) 13 g/l, creatinine kinase (CK) normal, while CRP was high (7 mg/l). Weakly positive anti-NOR 90 antibodies were found and noted to have unclear etiology with no clinical manifestation of scleroderma. Vitamin D deficiency was corrected (level: 47 nmol/L, normal range 50-150 nmol/L), and hypothyroidism medication was adjusted (TSH 7.7 IU/L, T4 9, normal range 12-22).

Despite extensive evaluations, including bilateral hands and feet X-rays, MRI of the hand, PET scan and laboratory assessments, the etiology of her symptoms remained elusive. Following a provisional diagnosis of fibromyalgia, the patient was managed symptomatically with medications, including pregabalin, amitriptyline, and duloxetine for one year. However, her symptoms persisted.

Further investigations revealed low serum cortisol levels: a morning cortisol level of 20 nmol/l (64-536), ACTH <0.3 pg/ml (1.6-13.9), and a 24-hour urine cortisol level of 11 nmol (28-138 nmol). Dual-energy X-ray absorptiometry (DEXA) scan demonstrated low bone mineral density with highest value at the lumbar sites (L2-L4), with a T-score of -2.4. Upon detailed review, it was noted that the individual had a history of frequent injections in both sacroiliac and lumbar facet joints, as well as trigger point injections ranging from 80-120 mg, administered every two to three months over a period of two years. Given the overall picture, with adequate adrenal response to synacthen test (the synacthen test results were as follows: baseline ACTH level was 1.2 pmol/L, rising to 0.8 pmol/L at 30 minutes and 0.4 pmol/L at 60 minutes; corresponding cortisol levels were 52 nmol/L at baseline, increasing to 433 nmol/L at 30 minutes and 472 nmol/L at 60 minutes), this was correlated with the diagnosis of iatrogenic Cushing's syndrome.

A summary of the cases is in Table 1, and the timeline of the cases is in Table 2.

**Table 1:** Summary of patients with Cushing syndrome who presented with fibromyalgia F: female, HTN: Hypertension, DM: Diabetes Mellitus, Rx: Treatment, ACTH: Adrenocorticotropic hormone

Case	Age	Gender	BMI	Steroid (Exogenous vs Endogenous)	HTN	DM	Hyperlipidemia	Psychiatric symptoms	Fracture	Abnormal Test Results	Treatment
Case 1	38	F	31.4	Endogenous- adrenal adenoma	Yes	No	No	No	Four fractures	Low potassium, low ACTH (<1pg/mL), high serum cortisol (604.03 nmol/L)	Adrenal adenoma surgical resection
Case 2	48	F	26	Endogenous- adrenal adenoma	Yes	Yes	Yes	Depression on Rx	Six fractures	Low ACTH (<0.3 pmol/L), high serum cortisol (1104 nmol/L), 24-hour urine free cortisol is high = 483 nmol (28-138)	Adrenal adenoma surgical resection
Case 3	35	F	38	Exogenous	Yes	No	No	Depression and anxiety on Rx	-	Low serum cortisol 20 nmol/l (64-536), low ACTH <0.3 pg/ml (1.6-13.9), 24-hour urine cortisol 11 nmol (28-138).	Refrain from injection

**Table 2:** Timeline of the three cases

Case	Timeline of clinical features	Final diagnosis date
Case 1	Bruises, myalgia, body pain since 2016; headache, body swelling since 2020; hypertension since 2021; hip pain since Jan 2022; fractured toe in Nov 2022; fracture of pubic rami discovered incidentally in April 2023; avascular necrosis of right hip in April 2023	May 2023 she was diagnosed with Cushing syndrome due to adrenal adenoma
Case 2	Widespread muscle and joint pain in 2017; hypertension and type 2 diabetes mellitus in 2019; multiple fractures in 2020-2021; anxiety, insomnia, and major depressive illness in 2020; menstrual irregularities in July 2021	November 2021 she was diagnosed with Cushing syndrome due to adrenal adenoma
Case 3	Polyarthralgia, proximal muscle weakness, profound fatigue, and peripheral oedema in 2021-2023; depression and anxiety in 2022; hypertension in 2023; low bone mineral density in 2023	June 2023 exogenous Cushing syndrome

## Discussion

Fibromyalgia is a multifactorial painful body disorder with several hypotheses regarding its etiology and pathophysiology such as increased pain sensitivity, neuroendocrine axis dysregulation, hypermobile joints, poor physical fitness, as well as genetic predisposition and environmental triggers [3].

Fibromyalgia and Cushing's syndrome are distinct medical conditions, but they can share some common symptoms such as fatigue, muscle weakness, mood changes, sleep disturbances, and memory deficits. Because of the multiple symptoms that are present in both, a patient could be misdiagnosed with fibromyalgia instead of Cushing’s syndrome if proper history-taking, physical examination and relevant investigation are not pursued. Fibromyalgia is a diagnosis of exclusion, so effort should be made to look for any possible cause of the patient’s symptoms before making a diagnosis of fibromyalgia. According to the American College of Rheumatology, a patient must satisfy these three conditions to be diagnosed with fibromyalgia: widespread pain index (WPI) ≥7 and symptom severity (SS) scale score ≥5 or WPI 3-6 and SS scale score ≥9, symptoms have been present at a similar level for at least three months, and the patient does not have a disorder that would otherwise explain the pain [4].

According to the 2008 Endocrine Society guidelines, Cushing syndrome’s diagnosis is made by lab tests that show consistently high production of cortisol using 24-hour urine free cortisol level, low-dose (1mg) dexamethasone suppression test, or late-night salivary or serum cortisol [5].

A literature review was performed using PubMed and Google Scholar databases. Search terms included “fibromyalgia” and “Cushing’s syndrome" to which five results were shown. Out of the five results, only one case report had slight relevance to our two cases which was about a 39-year-old woman previously diagnosed with Cushing’s disease who developed fibromyalgia [1]. Unlike our cases, she was already diagnosed with Cushing’s disease. Several cases of iatrogenic Cushing’s syndrome are widely recognized [6-10]. Although intra-articular corticosteroid injections are uncommon causes, they are becoming increasingly recognized especially in patients who have received multiple or relatively high doses [11-13].

Our patients saw different physicians from various specialties and had multiple hospital visits over two to three years. They were originally diagnosed with fibromyalgia. Despite a multitude of other symptoms and signs such as fractures, weight gain, amenorrhea, easy bruising, and hypertension, the initial diagnosis of fibromyalgia was carried forward by multiple physicians without proper re-evaluation, resulting in only symptomatic treatment. These cases highlight the importance of thorough clinical evaluation and a holistic approach to patients who present with fibromyalgia symptoms even if a previous diagnosis of fibromyalgia has been made. 

## Conclusions

These cases underscore the challenges in differentiating Cushing's syndrome from other conditions, particularly when presenting with nonspecific symptoms similar to fibromyalgia. Heightened clinical suspicion, thorough evaluation, and consideration of medication histories are essential. A high index of suspicion, combined with targeted radiological and biochemical testing, is crucial for accurate diagnosis and effective management.
